# Firearm Acoustic Detection in Hartford, Connecticut: Outcomes of a Trauma Center – Law Enforcement Collaboration

**DOI:** 10.7759/cureus.18789

**Published:** 2021-10-14

**Authors:** Brendan R Gontarz, Usman T Siddiqui, Brendan Campbell, Jonathan Gates, John Michael O'Hare, Christa Green, Jacqueline McQuay, David S Shapiro

**Affiliations:** 1 Surgery, University of Connecticut, Hartford, USA; 2 Surgery, Trinity Health of New England, Hartford, USA; 3 Pediatric Surgery, Connecticut Children's Medical Center, Hartford, USA; 4 Trauma, Hartford Hospital, Hartford, USA; 5 Criminal Justice, Hartford Police Department, Hartford, USA; 6 Surgery, Critical Care, Palliative Care and Trauma, Saint Francis Hospital (Trinity Health of New England), Hartford, USA

**Keywords:** shotspotter, general trauma surgery, trauma, gun-shot wound, prehospital emergency medicine, acoustic detection, gunfire

## Abstract

Introduction

Firearm homicide is a leading cause of violence-related death in the United States.Unfortunately, more than 80% of illegal firearm discharges are never reported to police by traditional means.ShotSpotter^TM^ (Newark, California) is an acoustic firearm event detection system that can localize gunfire, prompting police, and subsequent emergency medical services (EMS) presence. Previously reported healthcare effects of acoustic detection are speculative in nature. We sought to investigate Hartford, Connecticut's experience with ShotSpotter​​​​​​​^TM ^given its smaller size and broad coverage.

Methods

The three trauma centers in Hartford (two for adults and one for pediatric) collaborated with the Hartford Police to review outcomes of victims with acoustically detected gunshots and compare them to those who went undetected. We performed a retrospective review of patients who presented with gunshot wounds (GSW) over a 30-month period, from January 1, 2016 to June 30, 2018. Victim location and acoustic detection were reconciled by the police department and hospital staff independently. Patients were individually matched for location, prehospital response, treatment durations, and hospital outcomes.

Results

Of 387 GSW, 157 (40.6%) presented via EMS and were included in the sample. Of these, 89 correlated to a detection event (56.7%) and 68 had no correlating event (43.3%). These two groups had no difference in prehospital treatment times, scene and transport duration, and injury severity. Further, the need for surgery or transfusion, lengths of stay, and disposition, including mortality, did not differ.

Conclusions

Despite limited previous reports demonstrating conferred benefits to acoustic detection of gunshots, Hartford’s experience showed no benefit. The potential for such systems to act as early warning systems is evident but may depend on a city’s resources, geography, and technology.

## Introduction

Firearm homicide is a leading cause of violence-related death in the United States [1]. Although victims of firearm injuries have a high case-fatality rate, prompt first responder and prehospital responder attention have been demonstrated to improve survival [2-4]. Urban settings can offer swift access to trauma care, conferring a survival benefit compared to more rural environments. [5, 6]. Rapid transport, including police-involved transport, has also been shown to improve outcomes [2-9].

Locating a firearm victim can be challenging as more than 80% of illegal firearm discharges are never reported to police by local witnesses [1, 10]. ShotSpotter^TM^ (Newark, California) is an acoustic firearm event detection system (ADS) implemented by municipalities and university campuses to identify and localize firearm discharges [11]. Its ADS can localize gunfire to within 25 meters from where it occurred, and is less subject to variability from “earwitness” reports. ShotSpotter​​​​​​​^TM^ is a proprietary system that uses sophisticated acoustic sensors to detect, localize, and alert law enforcement or other authority about gunfire incidents in real-time. Acoustic sensors are strategically placed within a geographical distribution intended to be surveilled. Once identified, trained acoustic analysts qualify these highlighted incidents and confirm gunfire, reporting back to local authorities. This process usually takes no more than 45 seconds from the time of the actual event to the digital alert and localization on a map [11]. Standard police processes are utilized to respond to the gunfire, and if a victim is discovered upon arrival at the scene, emergency medical services (EMS) are deployed. The potential advantage of acoustic detection is that of an early warning system.

In 2012, Hartford, Connecticut obtained ShotSpotter​​​​​​​^TM^ to enhance firearm detection within the city limits. As of 2016, the system covered all residential areas and schools in the city. The healthcare effects of an ADS have been previously investigated in Oakland, California and Camden, New Jersey [12, 13]. Both investigations suggested a potential survival benefit when gunfire was captured by the ADS [12, 13]. There is significant disparity, however, between cities regarding their EMS, police, and trauma service resources. Violence disproportionately occurs in urban settings with active areas or “hot spots” where the bulk of violence occurs [1, 2]. Hartford, Connecticut is no exception. According to the Federal Bureau of Investigation (FBI)’s 2018 Uniform Crime Report, Hartford ranked 15th of the 40 most violent US cities [1]. Compared to previously studied ShotSpotter​​​​​​​^TM^ locations, Hartford is larger in square kilometers and population than Camden, NJ, but smaller than Oakland, California. Conversely, Hartford’s ADS has greater coverage than Oakland, but less coverage than Camden. Although Oakland is the largest of the three cities, it has two level 1 trauma centers (one pediatric, one adult), whereas Hartford has three (one pediatric and two adult). Unlike Camden, Hartford does not routinely transport injured patients by police vehicles. The inherent policing value of ADS notwithstanding, we hypothesized that Hartford’s smaller geographic size and increased proximity to trauma care offers uniform prehospital transport and that ADS-associated gunshot victims may not confer the same potential advantage with regards to victims’ outcomes.

## Materials and methods

In collaboration with the Hartford Police Department (HPD), we performed a retrospective review of patients who presented with gunshot wounds (GSW) to each of the city’s three trauma centers between January 1, 2016 and June 30, 2018. Patients who presented after a GSW within the city of Hartford, transported by EMS, and age 0-99 were included. All three hospitals are American College of Surgeons (ACS) verified Level I trauma centers. Medical records were meticulously reviewed, including EMS reports, patient interventions, and outcomes. Patients with firearm injuries were reviewed in two groups, those whose injuries correlated with a ShotSpotter^TM^ event (Acoustically Detected Events [ADE]) and those whose injuries were not associated with a detection event (No Acoustic Detection [NAD]). Criteria for reconciliation included matching of both time and gunfire location and were reconciled by the police department and hospital staff independently. Patients were individually matched for date and time of ADS event and location, EMS site location, and separately reconciled by HPD for site of ADS event and patient identity. Prehospital treatment times included response interval, scene arrival, duration at scene, transport time and overall prehospital time (defined as time from EMS activation to hospital arrival), were collected. Patient data included demographics, and outcomes including distance to hospital, length of stay (LOS), injury severity score (ISS), transfusions, operative interventions, mortality, and hospital discharge disposition. ShotSpotter^TM^ ADS event data was provided by HPD. Data was analyzed using SAS v9.4TM (SAS Institute, Cary, NC). Chi-squared was used to compare operative interventions and discharge disposition, and mortality, and Student’s t-test was used to compare prehospital times, transfusion requirements, and hospital lengths of stay. The primary outcome measure for this study is the time spent from injury to hospital arrival. The portions of prehospital times, outcomes of injury, and morbidity and mortality endpoints are also considered clinically important.

## Results

Over the 30-month study period, a total of 2562 gunfire events were recorded by the ShotSpotter^TM^ ADS. This total represents all-acoustic detections and all other witnessed or department call events. During the same period, 387 people presented to the study hospitals after sustaining a GSW. Of these patients, 157 (40.6%) presented via EMS within the city of Hartford and were included in our analysis. The remaining (230, 59.4%) presented by private vehicle or walk-in and were not involved in the analysis as EMS was not associated with their events.

Of the 157 patients in the study population, 89 correlated to a ShotSpotter^TM^ event (ADE, 56.7%), and 68 had no correlating event (NAD, 43.3%). ADE patients had an average prehospital time of 19.3 minutes, compared to NAD patients with an average of 18.8 minutes (p = 0.468). Total EMS scene time was similar, with 9.5 minutes for ADE vs. 9.3 minutes for NAD (p = 0.866).

In the patients presenting with GSW, police alerted EMS in 100% of cases. At the time of this investigation, acoustic detection data is not pushed to EMS personnel, thus EMS relies on police communication to identify patients in the field.

ISSs were similar between groups, with ADE patients' median ISS of 13.67 compared to NAD median ISS of 15.16 (p = 0.666). The groups were also similar with respect to the need for operative intervention, 29/89 (32.6%) ADE patients vs 25/68 (36.8%) NAD patients (p = 0.292). Additionally, the groups had similar blood product transfusion requirements with ADE patients receiving an average 2.3 units of packed RBCs (PRBC), 0.8 units of fresh frozen plasma (FFP), and 0.3 platelets (PLT), compared to NAD with 3.1, 1.8, and 0.7 units, respectively (Table 1).

**Table 1:** Prehospital times, interventions and outcomes, victims of gunshots in Hartford. km: Kilometers; EMS: Emergency medical services; PRBC: Packed red blood cells; FFP: Fresh frozen plasma; PLT: Platelets; LOS: Length of stay; DC: Discharge

	Detected Event (SD)	No Detected Event (SD)	
			p-value
Gunshot Injury Victims (n = 387)	89 (56.7%)	68 (43.3%)	
Distance from Hospital (km)	2.30 (1.03)	2.49 (1.66)	0.378
Age (SD)	29.84 (9.90)	31.05 (9.77)	0.447
% Male	89.9% (80)	91.2% (62)	0.392
Prehospital Time			
Prehospital Time	19.3±5.8	18.8±6.3	0.468
Total EMS Scene Time	9.5±4.0	9.3±4.2	0.866
Treatments			
Operative Interventions (#)	29/89 (32.6%)	25/68 (36.8%)	0.292
Transfusions (units PRBCs)	2.3±5.8	3.1±10.2	0.321
Transfusions (units FFP)	0.8±3.6	1.8±8.6	0.224
Transfusions (units PLT)	0.3±0.9	0.7±3.8	0.223
Outcomes			
Hospital length of stay	4.1±10.5	3.5±7.9	0.705
DC to rehabilitation	10 (11.2%)	7 (10.3%)	0.429
DC to home or police custody	66 (74.2%)	48 (70.6%)	0.308
Injury Severity Score (Median [SD])	13.67 [2.24]	15.16 [2.62]	0.666
Mortality	13 (14.6%)	13 (19.1%)	0.226

Hospital LOS between groups were also similar, with total LOS for ADE 4.1 days vs. 3.5 days for NAD, (p = 0.705). Patient disposition at discharge was similar with predominately home/police custody, ADE 66 or 74.2% vs. NAD, 48 or 70.6% (p = 0.308); followed by rehabilitation facility, ADE 10 or 11.2% vs. NAD: n = 7 or 10.3%, (p = 0.429). Mortality was also similar ADE 13 (14.6%) vs. NAD 13 (19.1%), (p = 0.226). Average distances to the hospital were also similar, with ADE patients at 2.30 kilometers from the hospital destination vs. NAD patients at 2.49 kilometers (p = 0.378).

## Discussion

This study demonstrated no significant difference in prehospital times, scene time, or transport duration for patients who sustained GSWs when acoustic detection occurred compared to patients who experienced GSW when no acoustic detection occurred. The previous reports did find a difference in prehospital time in those associated with acoustic detection showing improved transport times but are geographically distinct [12, 13]. The initial report out of Oakland, CA from Beattie G et al. found their ShotSpotter^TM^ event-matched patients had a higher median ISS, longer hospital LOS, more ventilator days, and were more likely to require surgical intervention, but overall mortality did not differ [12]. This suggests that more severely injured patients had improved survival with some attribution to the ADS, allowing for faster presentation to hospital care. In Camden, Goldenberg A et al. demonstrated decreased prehospital times when ShotSpotter^TM^ was activated in a city where police transport is possible [13]. These patients were also more severely injured, more likely to receive blood products, and had lower mortality, which suggested that the early interventions and transport conveyed a potential survival benefit [13]. Unlike Camden, Hartford does not routinely transport injured patients by police vehicles. While outside the scope of this study, further analyses could determine if transport by police vehicles would affect patient outcomes in our city. City size and ShotSpotter^TM ^coverage are depicted in Table 2. 

**Table 2:** Characteristics of cities employing ShotSpotter.

	Camden, NJ	Hartford, CT	Oakland, CA
Total area (sq km)	26.78	46.8	201.66
ShotSpotter Coverage (sq km)*	18.13	29.14	41.44
% of the total area covered	67.7	62.2	20.6
*Provided by the Hartford Police Department			

Previous studies of ADS in other cities made little distinction between areas included or excluded from acoustic detection. Hartford has the entirety of residential, educational, and corporate areas covered, and excludes only larger areas of special use (airport and firing range). Hartford is more similar to Camden in city coverage, and more similar to Oakland with respect to transport policy, rendering these cities not directly comparable. 

The extent of geographic acoustic detection coverage may be an important variable to consider, warranting additional study, especially in the policing literature. ADS use by police has been previously reported [2, 10-13]. ADS impact in gunshot survival may be less significant in smaller cities or those with sufficient trauma care resources, particularly in relation to the gun violence “hot spots” as demonstrated here [10-13]. While the utility of ADS coverage for an entire municipality may act as a deterrent to gunplay, the impact on healthcare remains mixed [10-13].

There is limited evidence on how real-time acoustic detection of gunfire may influence clinical outcomes for victims of GSWs [12, 13]. Variability in prehospital processes, resources, and geography among municipalities with ADSs may limit the ability to accurately make comparisons between cities. In theory, an “early warning system” which activates law enforcement and EMS services could allow for the earlier identification of GSW victims, allowing for more rapid transport times to trauma centers and improved clinical outcomes. This hypothesis, however, may not hold true if detection coverage is limited to less than a critical but undetermined threshold. Smaller cities with ease of access to trauma centers may find no benefit if transport time to hospitals is unvaried, especially when scene deaths are not included in the review. Larger cities with partial acoustic detection coverage may only find value when particular “hot spots” of violence are notably remote from the hospitals, but this data has yet to be discovered.

Since “scoop and run,” or the prioritization of rapid transport over on-scene resuscitation, is not practiced in Hartford, there is a possibility that early notification by acoustic detection would confer earlier activation of EMS and transport of patients for definitive care. Earlier notification could result in earlier arrival at the trauma center for these patients.

This study has multiple limitations. The retrospective nature of our study does not allow us to capture exactly how EMS was able to locate the patient. Whether location was identified by bystander phone call or law enforcement identifying the patient utilizing ADS and subsequently alerting EMS is unclear. EMS reports were reviewed, however, data was mostly limited to times, location, and brief patient encounter summaries. Police reports were not consistently available due to ongoing investigations. Many patients also present by private car or walk-in. Patients who present to the hospital directly are being assessed separately but are not directly comparable. These are challenging to reconcile with ADS events, as we must rely upon underreported patient-identified incident locations rather than relying on EMS data. Additionally, patient transport by private vehicle excludes initial care by first responders and EMS care and moves the site of initial therapeutic interventions to the hospital, which may not benefit from ADS, without police transport. Almost 60% of our GSW victims presented without EMS involvement. This likely stems from the fact that Hartford’s trauma centers are in very close proximity to its areas with the highest incidence of gun violence. This was confirmed by reviewing ShotSpotter^TM^ event maps. Patients who die at a scene associated with acoustic detection events were excluded as well.

Interestingly, only 89 (3.5%) of the 2562 activations over the 30-month period were associated with a patient presented to a study hospital via EMS. A total of 387 (15.1%) gunshot victims presented over the same period, regardless of presentation type or location of the injury event. With approximately 85% of firearm activations overall not included in the sample, there is room for additional acoustic detection review, including potential victims who did not seek medical attention, were deceased in the field, or those firearm events that were not associated with a victim. Though the ShotSpotter^TM^ system has a highly sensitive detection methodology, it does not detect most indoor firearm discharges, leaving victims in these locations potentially undetected. 

An acoustic firearm event detection system was not associated with shorter EMS transport times or improved patient outcomes for individuals who sustained GSW in the city of Hartford. Future studies evaluating ADS and firearm injury prospectively, and in further detail, will more accurately evaluate potential clinical benefits of ADS for victims of firearm-related violence. The potential for such systems to act as early warning systems is evident, but any benefit may be limited to municipalities with fewer trauma resources and maybe best in environments where police transport patients or where EMS is activated along with the police. While it may be premature to activate EMS upon acoustic detection alone, patterns in a particular city may suggest this deployment when considered with other factors and police cooperation.

## Conclusions

Although ADS may provide some benefit to victims of gun violence, this benefit is not universal. Cities with trauma centers located in close proximity to areas with high incidences of violent crime, particularly those without police transport policies, may not experience the same benefits of ADS in regards to trauma care. Individual cities should identify optimal utilization of police, EMS and trauma resources to tailor their prehospital care for victims of gun violence. Further research is needed to determine how to best incorporate ADS into prehospital trauma care.
